# Preoperative evaluation of accessory hepatic ducts by drip infusion cholangiography with CT

**DOI:** 10.1186/s12893-017-0251-9

**Published:** 2017-05-08

**Authors:** Hiromichi Ishii, Akinori Noguchi, Tomoyuki Fukami, Riho Sugimoto, Hiroyuki Tada, Hiroki Takeshita, Seiji Umehara, Hiroyuki Izumi, Naoki Tani, Masahide Yamaguchi, Tetsuro Yamane

**Affiliations:** Department of Surgery, Matsushita Memorial Hospital, 5-55 Sotojima-cho, Moriguchi city, Osaka, 570-8540 Japan

**Keywords:** Accessory hepatic duct, Drip infusion cholangiography with CT, Laparoscopic cholecystectomy, Bile duct injury

## Abstract

**Background:**

This retrospective study aimed to investigate the incidence of each type of accessory hepatic duct by drip infusion cholangiography with CT (DIC-CT).

**Methods:**

Five hundred sixty nine patients who underwent preoperative DIC-CT and laparoscopic cholecystectomy were reviewed. Accessory hepatic ducts were classified as follows: type I (accessory hepatic ducts that merged with the common hepatic duct between the confluence of the right and left hepatic ducts and the cystic duct confluence), type II (those that merged with the common hepatic duct at the same site as the cystic duct), type III (those that merged with the common bile duct distal to the cystic duct confluence), type IV (the cystic duct merged with the accessory hepatic duct), and type V (accessory hepatic ducts that merged with the common hepatic or bile duct on the left side).

**Results:**

Accessory hepatic ducts were observed in 50 patients. Type I, II, III, IV, and V accessory hepatic ducts were detected in 32, 3, 1, 11, and 3 patients, respectively. Based on their drainage areas, the accessory hepatic ducts were also classified as follows: a posterior branch in 22 patients, an anterior branch in 9 patients, a combination of posterior and anterior branches in 16 patients, a left-sided branch in 2 patients, and a caudate branch in 1 patient. None of the patients with accessory hepatic ducts suffered bile duct injuries.

**Conclusion:**

There are a number of variants of the accessory hepatic duct. DIC-CT is useful to detect the accessory hepatic duct.

## Background

Laparoscopic cholecystectomy (LC) has become the gold standard procedure for patients with cholecystolithiasis or gallbladder polyps; however, the incidence of bile duct injuries during LC has been reported to be about 0.5–1.7% [1, 2]. Anatomical anomalies of the biliary tree are considered to be a risk factor for bile duct injuries, and it was previously reported that the frequency of bile duct injuries is higher in patients with bile duct anomalies than in patients without them [3]. Therefore, it is important to evaluate the anatomy of the biliary tree, especially those of the cystic duct and any accessory hepatic ducts, preoperatively. Usually, drip infusion cholangiography with computed tomography (DIC-CT) or magnetic resonance cholangiopancreatography (MRCP) is performed to evaluate choledocholithiasis and the anatomy of the biliary tree before LC in Japan.

It was reported that accessory hepatic ducts exhibit an incidence rate of 2–11% [1, 4–7]; however, there are few reports about the drainage areas of accessory hepatic ducts.

The aims of this retrospective study are to investigate the incidence, type, and drainage area of accessory hepatic ducts using DIC-CT.

## Methods

### Patients

In total, 738 consecutive patients were scheduled to undergo LC for cholecystolithiasis, cholecystitis or gallbladder polyps at Matsushita Memorial Hospital between January 2004 and December 2015. DIC-CT was performed prior to elective LC for cholecystolithiasis, chronic cholecystitis or gallbladder polyps. Of these 738 patients, we investigated retrospectively the 569 consecutive patients who underwent preoperative DIC-CT in this study.

### DIC-CT examinations

One hundred milliliters of meglumine iotroxate (Biliscopin; BAYER, Osaka, Japan) were infused intravenously over a period of 30 min, and multidetector CT (Light Speed Ultra; GE Healthcare, Waukesha, USA) was performed 45 min later. Three-dimensional images were reconstructed using a surface-rendering method.

### Analysis of DIC-CT images

The bile ducts and cystic duct were evaluated using both two-dimensional and three-dimensional images. The bile duct located on the duodenal side of the bifurcation of the main portal vein was defined as the “main” extrahepatic bile duct, and intrahepatic bile ducts that joined the “main” extrahepatic bile duct were defined as accessory hepatic ducts. The drainage area of each accessory hepatic duct was evaluated using two-dimensional DIC-CT images. Patients involving poor visualization of the secondary branches of the intrahepatic bile ducts and/or the cystic duct were included in the poor study group.

**Fig. 1:**
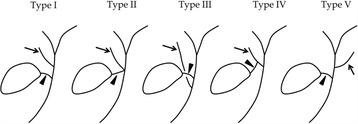
Classification of accessory hepatic ducts. The *arrow* indicates the accessory hepatic duct, and the *arrow head* shows the cystic duct

Accessory hepatic ducts were classified according to the method described in previous reports [1]: type I (accessory hepatic ducts that merged with the common hepatic duct between the confluence of the right and left hepatic ducts and the cystic duct confluence), type II (those that merged with the common hepatic duct at the same site as the cystic duct), type III (those that merged with the common bile duct distal to the cystic duct confluence), type IV (cases in which the cystic duct merged with the accessory hepatic duct), and type V (accessory hepatic ducts that merged with the common hepatic or bile duct on the left side). Accessory hepatic ducts that merged with the gallbladder and cases involving two accessory hepatic ducts were classified as “others” (Fig. 1). The accessory hepatic ducts were also classified according to their drainage areas as follows: the posterior branch type (accessory hepatic ducts that drained all or part of the right posterior section), the anterior branch type (those that drained all or part of the right anterior section), the combined posterior and anterior branch type (those that drained all or part of the right posterior section and all or part of the right anterior section), the left-sided branch type (those that drained part of the left hemiliver), and the caudate branch type (those that drained part of the caudate lobe).

### Surgical technique

LC was performed using four surgical trocars, and the critical view of safety technique is adopted for almost all patients. Intraoperative cholangiography was not carried out routinely. LC was converted to open cholecystectomy in cases involving severe adhesion around the gallbladder, bile duct injuries, or uncontrolled bleeding.

## Results

Of a total of 738 consecutive patients who underwent LC, DIC-CT was performed in 569 patients, MRCP was conducted in 94 patients, endoscopic retrograde cholangiography was carried out in 34 patients, cholangiography was performed using a percutaneous transhepatic gallbladder drainage tube in 3 patients, and no examination of the biliary tree was conducted in 38 patients. Of the 569 patients that underwent DIC-CT, 11 belonged to the poor study group.

**Fig. 2:**
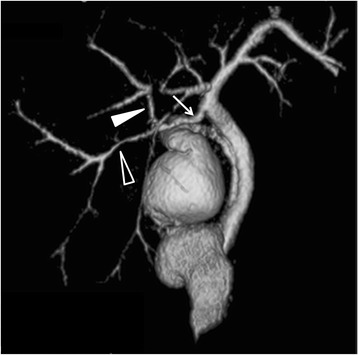
Three-dimensional DIC-CT (right ventral view) showing a type I accessory hepatic duct. The accessory hepatic duct (*arrow*) drained both the dorsal area of the right anterior section (*white arrow head*) and the right posterior section (*black arrow head*) and was classified into the combined posterior and anterior branch type

**Fig. 3:**
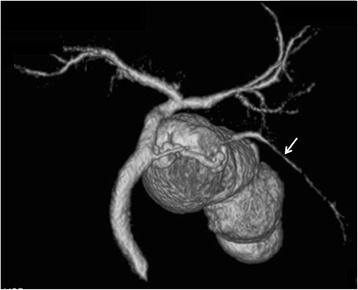
Three-dimensional DIC-CT image (dorsal view) showing a type II accessory hepatic duct. The accessory hepatic duct drained segment 6 (*arrow*) and was classified as belonging to the posterior branch type

**Fig. 4:**
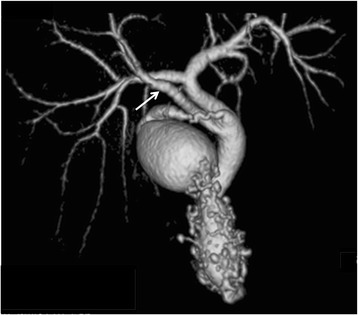
Three-dimensional DIC-CT image (ventral view) showing a type III accessory hepatic duct. The accessory hepatic duct drained the right anterior section (*arrow*) and was classified as belonging to the anterior branch type

**Fig. 5:**
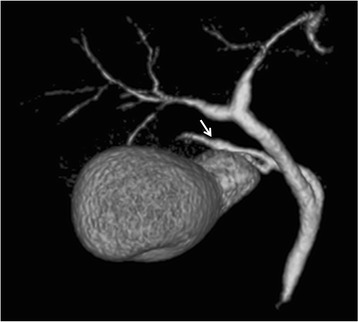
Three-dimensional DIC-CT image (ventral view) showing a type IV accessory hepatic duct. The accessory hepatic duct drained the right posterior section (*arrow*) and was classified as belonging to the posterior branch type

**Fig. 6:**
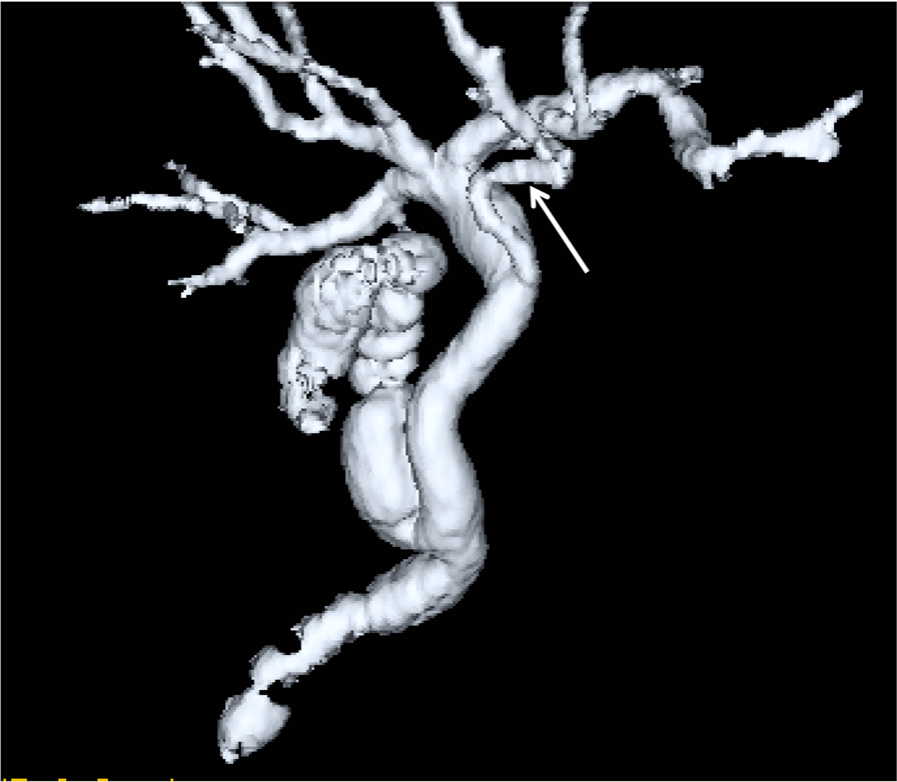
Three-dimensional DIC-CT image (ventral view) showing the type V accessory hepatic duct. The accessory hepatic duct drained segment 4 (*arrow*) and was classified into the left-sided branch type

**Fig. 7:**
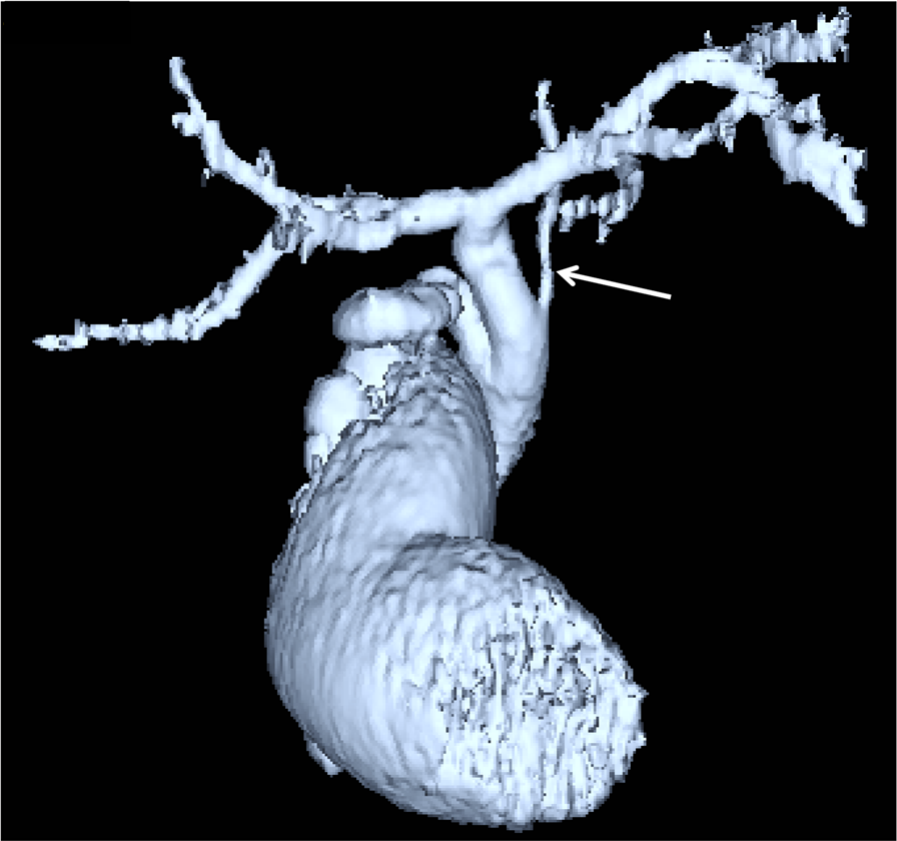
Three-dimensional DIC-CT image (ventral view) showing a type V accessory hepatic duct. The accessory hepatic duct drained the Spiegel lobe (*arrow*) and was classified into the caudate branch type

Fifty (9%) of the 558 patients that did not belong to the poor study group had accessory hepatic ducts (Table 1). Type I, II, III, IV, and V accessory hepatic ducts were observed in 32 (64%) (Fig. 2), 3 (6%) (Fig. 3), 1 (2%) (Fig. 4), 11 (22%) (Fig. 5), and 3 patients (6%) (Figs. 6, 7), respectively. The accessory hepatic ducts were classified as the posterior branch type in 22 patients (44%) (Figs. 3, 5), the anterior branch type in 9 patients (18%) (Fig. 4), the combined posterior and anterior branch type in 16 patients (32%) (Fig. 2), the left-sided branch type in 2 patients (4%) (Fig. 6), and the caudate branch type in 1 patient (2%) (Fig. 7). Following the separation of the gallbladder from the liver bed, the cystic duct was clipped and divided to prevent bile duct injuries in the patients with type IV accessory hepatic ducts.

**Table 1:** Variations in the types and drainage areas of accessory hepatic ducts

	Type I	Type II	Type III	Type IV	Type V	Others	Total
Posterior branch	16	1	0	5	0	0	22
Anterior branch	6	0	1	2	0	0	9
Combined branch	10	2	0	4	0	0	16
Left-sided branch	0	0	0	0	2	0	2
Caudate branch	0	0	0	0	1	0	1
Total	32	3	1	11	3	0	50

Although bile duct injuries occurred in 4 (0.7%) of the 569 patients that underwent LC, none of the patients with accessory hepatic ducts suffered bile duct injuries. All of the bile duct injuries were noticed during the operation, and primary repair of the injuries was performed via conversion to open laparotomy. Two patients underwent cholangioduodenostomy, and two patients underwent simple closure of their bile duct injuries. LC was converted to open cholecystectomy in 23 patients (4%) due to severe adhesion in 15 patients, bile duct injuries in 4 patients, uncontrolled bleeding in one patient, and other reasons in 3 patients. Conversion to open cholecystectomy was necessary in one of the 50 patients with accessory hepatic ducts due to severe adhesion.

## Discussion

DIC-CT or MRCP is usually performed preoperatively in patients who undergo cholecystectomy to evaluate choledocholithiasis and the anatomy of the biliary tree in Japan. Although MRCP is the most widely used non-invasive means of evaluating biliary disease, DIC-CT depicts small bile ducts (e.g., accessory hepatic ducts, the cystic duct, or the caudate branch) than MRCP [7, 8]. Because only 11 patients (1.9%) were classified in the poor study group in this study, DIC-CT is successful in assessing the biliary tree anatomy. Therefore, DIC-CT is performed routinely at our institution except in patients who exhibit adverse reactions to contrast media or severe thyroid disease. We consider that the radiation during DIC-CT does not affect the patient’s health because the radiation dose during DIC-CT is about 8 mGy. The side effects of meglumine iotroxate have been reported, however, severe side effects such as anaphylactic shock did not occur in this study.

The incidence of accessory hepatic ducts was reported to range from 2 to 11% in previous studies [1, 4–7], whereas it was 9% in this study. The incidence rates of type I, II, III, IV, and V accessory hepatic ducts were previously reported to range from 35 to 66.7%, 2–19%, 0–21.6%, 13–25%, and 0–7.7%, respectively [1, 4–7]. In this study, type I accessory hepatic ducts exhibited the highest frequency, followed by type IV ducts. Types II, III, and V were less common, as was found in previous studies. On the other hand, there have been few reports about the drainage areas of accessory hepatic ducts. It was reported that most accessory hepatic ducts (86.7–100%) are right posterior bile ducts [1, 6]. Hirao et al. [7] found that of 13 patients with accessory hepatic ducts 9 (69%) had posterior ducts, two had anterior ducts (15%), one had an anteroinferior branch (8%), and a caudate branch was seen in the remaining patient (8%). In our study, we investigated the drainage areas of accessory hepatic ducts in detail. Although the posterior branch type exhibited the highest incidence (44%), several other types of accessory hepatic ducts, such as the combined posterior and anterior branch type (32%), anterior branch type (18%), left-sided branch type (4%), and caudate branch type (2%) were also seen. This information is important not only for cholecystectomy, but also for surgery for cholangiocarcinoma [9, 10], pancreatic head tumors [11] and for transplant procedures involving living liver donors [12–14].

The causes of bile duct injuries during LC can be divided into anatomical [3, 15], inflammatory [16], and technical factors. The critical view of safety [17] is the important technique for preventing bile duct injuries during LC. Several authors have reported that intraoperative cholangiography reduces bile duct injury [18, 19], on the other hand, it has been done that intraoperative cholangiography does not reduce bile duct injury [20], and that injuries to accessory hepatic ducts or anomalous cystic ducts cannot be prevented by intraoperative cholangiography because anomalous bile ducts sometimes cannot be found with intraoperative cholangiography [4]. It has been reported that fluorescence cholangiography is a safe and effective procedure that enables real-time visualization of the biliary tree, and this novel procedure may become standard practice in order to prevent bile duct injury [21]. In our study, all of the bile duct injuries occurred in patients with severe inflammatory adhesion due to chronic cholecystitis. Thus, even in patients with accessory hepatic ducts, precise preoperative diagnosis and the meticulous performance of surgical procedures might prevent bile duct injuries. Of the various types of accessory hepatic ducts, type I, II, III, and IV ducts, which were observed in 32 (5.7%), 3 (0.5%), 1 (0.2%), and 11 patients (2.0%), respectively in this study, are at risk of bile duct injuries during the Calot triangle dissection. Especially, type IV ducts carry the greatest risk of bile duct injuries [1, 5, 6]. Noji et al. [4] suggested that the installation of an endoscopic nasobiliary drainage tube prior to LC in cases involving predictable bile duct anomalies might reduce the incidence of complications, and Kurata et al. [6] did that bile duct injuries can be avoided by using a surgical technique that exposes the inner layer of the subserosal layer of the gallbladder wall. We consider that intraoperative fluorescence cholangiography may be useful procedure to avoid bile duct injuries when patients with type I, II, III, or IV accessory hepatic ducts have severe inflammatory adhesion due to chronic cholecystitis. Furthermore, we recommend the dome down technique, in which the cystic duct is divided following the separation of the gallbladder from the liver bed, to prevent bile duct injuries in patients with type IV accessory hepatic ducts. Type V accessory hepatic ducts do not usually cause problem during LC; however, surgeons should take special care to prevent injuries to accessory hepatic ducts in segment 2 (type V) in patients with true left-sided gallbladders [22].

## Conclusions

There are a number of variants of the accessory hepatic duct. Surgeons should pay special attention to accessory hepatic ducts to prevent bile duct injuries during LC. Furthermore, the knowledge of the variations in accessory hepatic ducts is important for hepatobiliarypancreatic surgery.

## Abbreviations

DIC-CT: Drip infusion cholangiography with computed tomography
LC: Laparoscopic cholecystectomy
MRCP: Magnetic resonance cholangiopancreatography
